# Editorial: Invasive Treatments for Obsessive Compulsive Disorder

**DOI:** 10.3389/fpsyt.2021.764003

**Published:** 2021-09-28

**Authors:** Marshall T. Holland, Nicholas T. Trapp, Jeremy D. W. Greenlee

**Affiliations:** ^1^Department of Neurosurgery, University of Alabama at Birmingham, Birmingham, AL, United States; ^2^Department of Psychiatry, University of Iowa, Iowa City, IA, United States; ^3^Department of Neurosurgery, University of Iowa, Iowa City, IA, United States

**Keywords:** neuromodulation, neurostimulation, neuropsychiatric disorder, deep brain stimualtion, obsessive Compulsive Disorder (OCD)

When we were asked to be guest editors on this special collection, we were excited to provide a forum to discuss the current state and potential future of surgical treatment of obsessive-compulsive disorder (OCD). One notable aspect of this topic is that it sits at the crossroads of several disciplines including, but not limited to neuroscience, neurosurgery, psychiatry, neurology, and medical ethics, with each providing valuable insights into the nature of the disease and its treatment.

OCD affects 1–2% of the general population with 10% of patients being refractory to pharmacologic and psychotherapeutic treatment with 40–60% achieving only partial response (1, 2). On *average* patients with chronic OCD reported spending ~10.5 h per day engaged with their disabling thoughts and behaviors, and two-thirds rate their role impairment as severe (3, 4). Furthermore, the patient population seeking and receiving procedural intervention often has the most severe and disabling symptoms, hoping for any relief to restore quality of life at a stage of illness when little else can be offered. However, there is reason to believe that the future of invasive therapy for OCD can offer therapy that is more than palliative. As our understanding of the pathophysiology and neurocircuitry involved in this disorder improves, so too will our procedural precision and clinical outcomes. This topic is explored in depth by Vieira et al.

Neuromodulation, with its benefits of being testable and reversible, has gained significant traction in this field, specifically deep brain stimulation (DBS). Despite its approval by the FDA in February of 2009 for Humanitarian Device Exception and evidence of improvement in multiple small series reports (5–7), DBS for OCD, in our opinion, remains woefully underutilized. The underpinning reasons for this are explored by Pinckard-Dover et al. as well as Davis et al..

As the field pushes forward with intensity exploring new surgical techniques, there is interest in less invasive techniques such as focused ultrasound as presented by Chang et al. Most prominently used for tremor, we are interested in seeing the evolution and use of focused ultrasound for a variety of indications, including long term studies with OCD. We will follow this development with earnest.

An exciting and growing field is the procedurally inclined interventional psychiatry. As evidence grows for the effectiveness of procedure-based treatments for neuropsychiatric illnesses, a growing number of psychiatrists are embracing invasive interventions, particularly neuromodulation (8, 9). How to educate, train and grow this field further is discussed by Trapp and Williams.

We hope this collection will provide the reader with a framework for thinking about the invasive approach to treatment of refractory OCD. We believe the variety of topics discussed, and the diversity of disciplines represented in this collection of works demonstrates the necessity and promise of a multidisciplinary approach. We also hope this work will inspire readers to pursue further research which will lead to breakthroughs that may be translated to therapies to improve the quality of life of our patients.

## Author Contributions

MH wrote the first draft of the editorial. MH, NT, and JG critically revised the manuscript and approved the final submission. All authors contributed to the article and approved the submitted version.

## Funding

Dr. NT's time is supported by grant NIMH K23MH125145.

## Conflict of Interest

The authors declare that the research was conducted in the absence of any commercial or financial relationships that could be construed as a potential conflict of interest.

## Publisher's Note

All claims expressed in this article are solely those of the authors and do not necessarily represent those of their affiliated organizations, or those of the publisher, the editors and the reviewers. Any product that may be evaluated in this article, or claim that may be made by its manufacturer, is not guaranteed or endorsed by the publisher.
